# Monaural Beamforming in Bimodal Cochlear Implant Users: Effect of (A)symmetric Directivity and Noise Type

**DOI:** 10.1371/journal.pone.0160829

**Published:** 2016-08-18

**Authors:** Elke M. J. Devocht, A. Miranda L. Janssen, Josef Chalupper, Robert J. Stokroos, Erwin L. J. George

**Affiliations:** 1 Department of ENT/Audiology, School for Mental Health and Neuroscience (MHENS), Maastricht University Medical Center, Maastricht, The Netherlands; 2 Department of Methodology and Statistics, School for Public Health and Primary Care (CAPHRI), Maastricht University, Maastricht, The Netherlands; 3 Advanced Bionics European Research Centre, Hannover, Germany; Northwestern University, UNITED STATES

## Abstract

**Objective:**

To evaluate monaural beamforming in bimodally aided cochlear implant (CI) users.

**Design:**

The study enrolled twelve adult bimodal listeners with at least six months of CI-experience and using a contralateral hearing aid (HA) most of the daytime. Participants were uniformly fitted with the same CI speech processor and HA, giving access to an identical monaural beamformer in both ears. A within-subject repeated measures design evaluated three directional configurations [omnidirectional, asymmetric directivity (in CI alone) and symmetric directivity (in both CI and HA)] in two noise types [stationary and fluctuating]. Bimodal speech reception thresholds (SRT) as well as listening effort ratings were assessed in a diffuse noise field.

**Results:**

Symmetric monaural beamforming provided a significant SRT improvement of 2.6 dB SNR, compared to 1.6 dB SNR for asymmetric monaural beamforming. Directional benefits were similarly observed in stationary and fluctuating noise. Directivity did not contribute to less listening effort in addition to improvement in speech intelligibility. Bimodal performance was about 7 dB SNR worse in fluctuating than in stationary noise.

**Conclusions:**

Monaural beamforming provided substantial benefit for speech intelligibility in noise for bimodal listeners. The greatest benefit occurred when monaural beamforming was activated symmetrically in both CI and HA. Monaural beamforming does not bridge the gap between bimodal and normal hearing performance, especially in fluctuating noise. Results advocate further bimodal co-operation.

**Trial Registration:**

This trial was registered in www.trialregister.nl under number NTR4901.

## Introduction

Although most cochlear implant (CI) recipients can achieve high levels of speech intelligibility in quiet [1], understanding speech in the presence of noise or competing talkers remains a major challenge [2,3]. Useful input to both ears is a way to improve speech perception in noise. Both bilateral CIs and bimodal hearing, referring to the combination of a CI and a conventional hearing aid (HA) in opposite ears, are known to improve intelligibility in noise by offering access to bilateral and binaural cues [4–6]. CI candidacy criteria are expanding [7] and now include patients with aidable residual hearing. Bimodal fitting therefore has become well-established clinical practice [8] and higher bimodal hearing aid retention rates have been reported [9]. It has been shown that by combining modalities, the limits of electrical hearing can be complemented by low-frequency information retrieved from the acoustic ear [10–12].

An alternative approach to enhance speech intelligibility in noise is to improve the signal-to-noise ratio (SNR) before sound is offered to the ear. Given that interfering sources are often spatially separated, a directional microphone system may be applied to focus on the target speech while attenuating noise from other directions [13]. Creating microphone directionality is often referred to as *beamforming* [14]. Modern beamformers make use of multi-microphone arrays [15] located at the same (monaural) or across ear sides (binaural) [16] and can function in a fixed or adaptive manner [17]. Directional microphone systems, available in HAs since the 1970s [18], substantially improve speech intelligibility in noise [19–21]. Since 2005 also CI recipients have been able to benefit from monaural adaptive beamforming [22–29].

**Fig 1:**
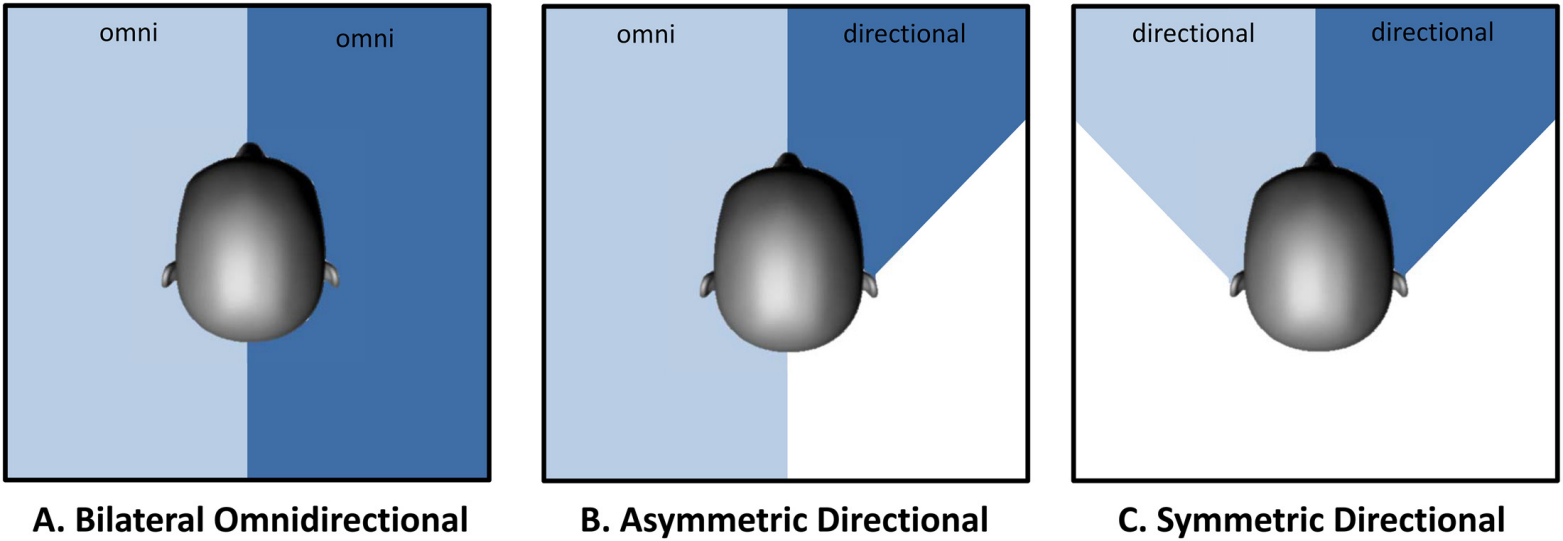
(A)symmetric directivity. Schematic illustration of bilateral omnidirectional (A), asymmetric directional (B) and symmetric directional (C) configurations.

Given that directional microphone systems are now available for both HA and CI, and that benefits provided by bimodal hearing are expected, it can be hypothesized that the two approaches are complementary in improving speech intelligibility in noise. An unaddressed question related to the bimodal application of directivity is whether to activate beamforming in both CI and HA or only in the primary speech input, which is often the CI. When a directional microphone is activated in one ear while an omnidirectional microphone is used in the other ear, it is referred to as an *asymmetric* directional fitting [30] (Fig 1). Given that an asymmetric hearing situation is often predefined when combining CI and HA in opposite ears, the evaluation of directional symmetry is of particular interest in bimodal hearing.

The masking efficiency of noise is known to depend on the availability of temporal gaps and fine structure, as well as the degree of perceptual similarity between target and interferer [31]. Speech-in-noise testing is traditionally performed in stationary noise in the absence of the above-mentioned characteristics. Non-stationary and modulated maskers are however considered as being more representative of everyday listening situations [32,33]. Bimodal users still have some limited access to low frequency hearing through the use of a contralateral HA. It could therefore be argued that acoustic HA use, to some extent, facilitates listening in the temporal gaps and using temporal fine structure which cannot occur via electric CI stimulation [34]. Moreover, directional microphones enhance the SNR available to the ear theoretically giving more access to temporal information of speech within background noise. Directional microphones could then, in particular, be expected to improve performance in fluctuating noise [24].

Measurements of speech perception in noise seldom extend beyond intelligibility. It is however known that in challenging auditory environments it can be ‘easier’ or ‘harder’ to listen to speech even for identical levels of intelligibility [35,36]. Background noise namely can make speech communication tiring and cognitively taxing, especially for individuals with hearing impairment [37]. Sometimes listening difficulty ratings can evaluate speech transmission more accurately and sensitively than intelligibility scores, especially at high performance levels [38]. Even though improvement in intelligibility has not been seen, noise reduction algorithms have proven to reduce listening effort [39] and free up cognitive resources for other tasks [40]. Also directional microphone systems have previously been described as easing listening effort in some HA studies [41,42].

The current study was designed to evaluate the performance of a monaural beamformer in bimodal listeners. It was hypothesized that, for speech perception in noise, symmetric directional fitting (monaural beamforming in both CI and HA) could provide more benefit than asymmetric fitting (monaural beamforming in CI only). Hence both directional configurations were compared to the omnidirectional standard for the primary outcome of speech intelligibility and the secondary outcome of listening effort in the presence of a stationary as well as a fluctuating masker.

## Materials and Methods

### Ethics

The study was approved by the local Medical Ethical Committee (Maastricht University Medical Center, NL51559.068.14), registered in the Dutch National Trial Register (NTR4901) and conducted in accordance with the Declaration of Helsinki. All bimodal participants provided written informed consent prior to participation and received compensation for participation and travelling expenses.

### Participants

The bimodal study group consisted of twelve adult bimodal listeners (8 male/4 female; mean age 64.6 years, SD 14.2 years, range 23–77 years). All participants were Dutch speaking and had at least 6 months of regular experience with a CI speech processor of the brand Advanced Bionics (AB) (Valencia, USA). In the contralateral ear all participants had to use a conventional HA for at least 50% of the daytime. The aided phoneme score in quiet for the bimodal situation had to be at least 50% to ensure ability to participate in speech-in-noise testing [43] and be included as a participant in the current study. Details on the individual hearing situation of the bimodal participants are presented (Table 1). To estimate the effect of noise type in the speech-in-noise test, a normal hearing group was considered (n = 7, age 27.3 years, SD 4.5 years, audiometric thresholds ≤20 dB HL) as reference.

**Table 1:** Participant characteristics.

		CI						HA					CI+HA
Subject	Etiology	Ear-side	Experience^1^	Processor	Strategy^3^	ClearVoice^3^	Word-score^4^	Experience^1^	Brand	Type	PTA^9^	Word-score^4^	Word-score^4^
**BZ01**	Hereditary	L	1.6	Naida CI Q70^2^	HiRes Optima-S	High	90	21	Phonak^5^	Solana SP	90.0	78	100
**BZ02**	Noise	L	1.4	Naida CI Q70^2^	HiRes Optima-S	High	80	23	Phonak^5^	Naida S III UP	80.0	53	95
**BZ03**	Meniere	R	2.4	Harmony^2^	HiRes-S/Fid120	Medium	78	29	Phonak^5^	Naida V UP	100.0	38	70
**BZ04**	Hereditary	R	0.9	Naida CI Q70^2^	HiRes Optima-S	High	90	11	Phonak^5^	Naida S V UP	63.3	84	88
**BZ05**	Meniere	R	1.6	Naida CI Q70^2^	HiRes Optima-S	High	65	2	Phonak^5^	Certena M	70.0	85	78
**BZ06**	Meniere	L	1.1	Naida CI Q70^2^	HiRes Optima-S	Medium	70	15	Sonic^6^	Endura 12 SP	85.0	9	78
**BZ07**	Unknown	R	8.5	Harmony^2^	HiRes-P/Fid120	Off	48	52	Oticon^7^	Ino Pro	88.3	52	62
**BZ08**	Turner syndrome	R	3.7	Harmony^2^	HiRes-S/Fid120	Off	66	48	Phonak^5^	Naida III UP	105.0	54	78
**BZ09**	Hereditary; Trauma	R	5.6	Naida CI Q70^2^	HiRes-S/Fid120	Off	73	20	Oticon^6^	Agil	85.0	58	70
**BZ10**	Unknown; Sudden deafness	R	1.5	Naida CI Q70^2^	HiRes Optima-S	High	90	13	Phonak^5^	Naida S V SP	65.0	50	90
**BZ11**	Otosclerosis	R	6.4	Naida CI Q70^2^	HiRes Optima-S	High	68	27	Siemens^8^	Nitro 701 SP	113.3	18	78
**BZ12**	Noise	R	3.2	Harmony^2^	HiRes-P/Fid120	High	76	18	Phonak^5^	Naida S I UP	90.0	39	91

CI = cochlear implant; HA = hearing aid; R = right; L = left;

^1^ expressed in years;

^2^ Advanced Bionics^™^ (Valencia, USA);

^3^ settings in the participants’ most used daily CI program;

^4^ aided maximum % correct consonant-nucleus-consonant (CNC) score in quiet free-field up to 75 dB SPL with participants' clinical fitting;

^5 ™^(Stäfa, Switserland);

^6 ™^(Bern, Switserland);

^7 ™^(SmØrum, Denmark);

^8 ™^(Erlangen, Germany);

^9^ unaided pure-tone average (PTA) across 0.5,1,2 kHz under headphones.

### Monaural beamforming

The UltraZoom^™^ system was used since this directional system is identically available in CI processors by AB [28,29] as well as in HA’s by Phonak due to the collaboration between these two manufacturers within the Sonova group (Stäfa, Switserland). This directional system is a monaural beamformer based on an array of two omnidirectional microphones. Sound attenuation for the back hemisphere is adaptively steered in a frequency specific manner depending on the noise source orientation [44].

### Device fitting

All participants were fitted in the context of the study with the exact same CI speech processor and HA in order to avoid variability of devices and microphone effectiveness [45].

The participant’s daily CI program (based on a HiRes Fidelity120^™^ or Optima^™^ processing strategy, Table 1), was transferred without changing basic map parameters from their everyday processor into the selfsame study speech processor (Naida CI Q70^™^,AB). The optionally activated noise reduction algorithm (ClearVoice^™^ [46,47], Table 1) was converted accordingly. No interaction exists between this algorithm and the investigated beamformer [29].

In the opposite ear, all participants were uniformly fitted with the same hearing aid (Naida Q90 UP^™^, Phonak) to their own closed earmold. The HA’s proprietary formula (Adaptive Phonak Digital) was used to calculate the prescribed acoustic gain based on the participant’s residual hearing thresholds. The optimized frequency response and aligned compression of the Bimodal Formula [48] was applied to enhance fitting for the bimodal situation. All other HA processing features, aside from feedback cancellation, were deactivated to avoid interactions. Following a short acclimatization period, gain settings were individually fine-tuned based on the participant’s feedback of interaural balance or comparability with their daily fitting.

### Study design

**Fig 2:**
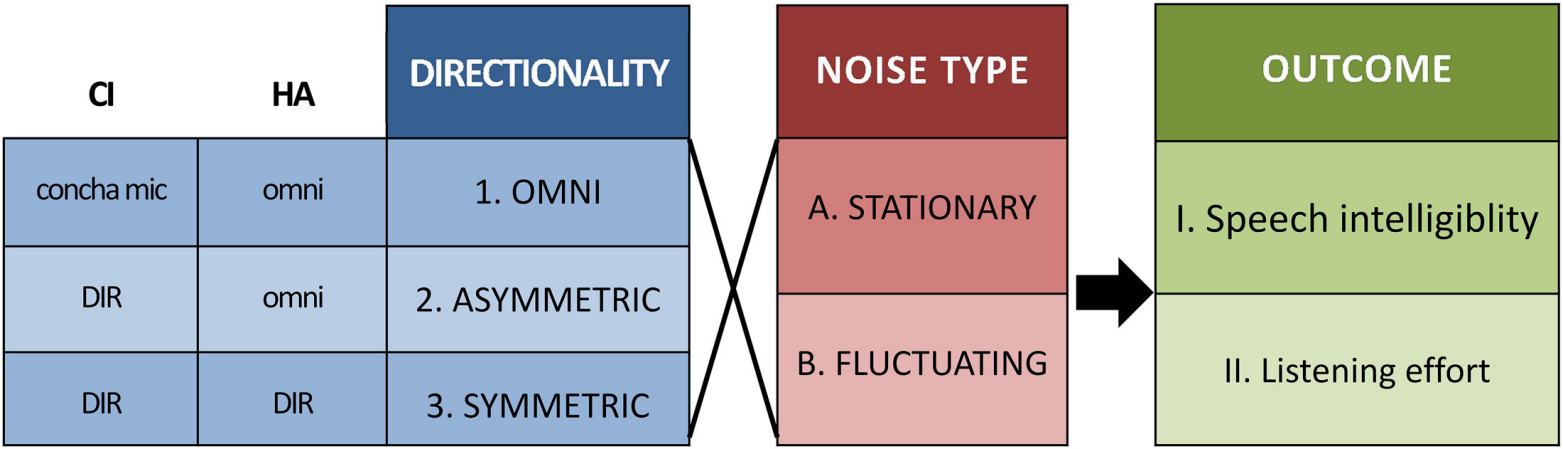
Study design. Two outcome measures in two types of noise were used to evaluate the effect of three directional configurations in users of a cochlear implant (CI) and a hearing aid (HA) in opposite ears. DIR refers to the application of a monaural adaptive beamformer.

A within-subject repeated measures design was applied (Fig 2). Speech intelligibility performance (I) was first assessed followed by listening effort (II). Each outcome measure was tested for the bimodal situation in three directional configurations (1,2,3) and two masking noises (A and B), resulting in six test conditions per outcome. To control for sequencing, test conditions were randomised across subjects for each outcome measure separately using a balanced Latin Square design.

#### Directional configuration

Three directional configurations were single-blindly evaluated: omnidirectional (1), asymmetric (2) and symmetric directivity (3). To switch and verify the directional configurations according to the condition to be tested, the researcher used a remote control on the CI as well as the HA. Participants were not informed about the directional settings of their devices during testing.

The reference condition was the omnidirectional setting with the standard microphones in CI and HA. For the HA the basic standard is an omnidirectional behind-the-ear microphone. For the CI processor, the default microphone is the T-Mic^™^: an omnidirectional microphone positioned in the pinna [49]. In the symmetric configuration, directivity (DIR) was switched on in the CI as well as the HA, resulting in the same monaural beamformer applied bimodally.

In order to keep total test time within participants’ concentration span, only the single most relevant asymmetric directional setting was included. For CI-recipients the CI-side in general is assumed to serve as the primary input for speech intelligibility. Furthermore it has been observed that severe hearing impaired listeners are less likely to use a directional microphone program on their HA [50] since they have a significantly lower potential in directional benefit when compared to listeners with moderate hearing loss [51]. Therefore the most clinically relevant asymmetric configuration of primary interest in this study consisted of activating the monaural beamformer (DIR) at the CI-side while keeping the HA at the omnidirectional setting.

#### Speech intelligibility

To assess speech intelligibility in noise (I), the optimized version of the Dutch Matrix test [52] was used. This speech-in-noise test is based on a closed speech corpus of sentences with the same fixed syntactical structure founded on five word categories ‘name, verb, numeral, adjective, object’. For example ‘ Mark gives five large flowers. ‘ More details on the Dutch Matrix test can be found in publications by Houben et al [52,53]. The corpus is by design well suited for repetitive testing and has proven to be applicable for use in cochlear implant recipients [43]. The test was administered as a closed-set. The participants provided responses on a digital touch screen displaying a matrix containing the ten alternative tokes within each of the five word categories. Since the use of an ‘I don’t know’-button was not allowed, the participant was forced to make a choice within each of the five categories to reconstruct the perceived sentence.

The noise was kept at a fixed overall level of 65 dB SPL, while the speech level started off at +5 dB SNR being adjusted subsequently in an adaptive procedure [54] based on word scoring. The procedure aimed at finding the signal-to-noise ratio (SNR) that yielded a sentence recognition score of 50% correct, defined as the speech-reception-threshold (SRT). To address potential learning effects [43] and familiarize participants with the task, two training lists of 20 sentences were administered (one for each type of noise) prior to the start of actual testing. The results of these training lists were excluded from analysis. To obtain a reliable indication of directional benefit [55] in each of the six test conditions, all conditions were assessed twice in a randomized order that was identical for test and retest. Each list consisted of 20 sentences and had a test time of 5 minutes on average. The sequence of lists was kept constant for all participants across randomized test conditions in order to prevent the usage of the same list twice within one participant. Overall this procedure resulted in 12 lists per participant (2 lists * 6 conditions). The actual speech-in-noise test had an average total duration of 60 minutes. To counteract fatigue, two intermissions were scheduled, one halfway through the speech-in-noise test and one before switching to the listening effort measurements. Additional breaks could be taken according to individual need.

When the adaptive procedure led to an invalid SRT outcome, defined as a SNR result outside the range of presented levels or above 15 dB SNR [56], the outcome was omitted. If there were two valid outcomes, the final result per condition was calculated as the mean of test and retest.

#### Listening effort

Participants were asked to rate the effort it took to listen to sentences in noise for the listening effort test (II). Rating was performed using a vertical scale with 13 discrete points (7 named categories interspersed by an empty category) ranging from ‘no effort’ (score 0) to ‘extreme effort’ (score 12) [57]. The corpus of unique sentences from the Dutch Matrix test [52] was also used for effort rating. Noise was presented at a fixed overall level of 65 dB SPL. In order to evaluate listening effort on top of speech intelligibility, the level of speech was set at the participant’s individual SRT outcome in the speech intelligibility test for the corresponding condition. Listening effort was assessed at three levels: the participant’s individual SRT, 5 dB above (SRT+5) and 10 dB above SRT (SRT+10). Every time a level was presented, one randomly selected sentence was repeated until the participant was confident enough to provide a rating. After a practice run with each level presented only once, every level was presented five times and the result was calculated as the mean of these five ratings. All six test conditions were assessed in random order with an average total test time of 12.5 minutes.

#### Noise type

Both outcome measures were assessed in two maskers: stationary (A) and fluctuating (B) noise. The default stationary noise associated with the Dutch Matrix [53] test was applied. This noise is composed of a randomly-aligned superposition of all the sentences in the test corpus and therefore has the same average power spectrum as the speech material [53]. As fluctuating noise masker, a modification of the International Female Fluctuating Masker (IFFM) [58] was applied. The IFFM consists of a multilingual voice signal that has the spectral and temporal characteristics of a single speaker but is non-intelligible as a whole. The modification consisted of decreasing the fundamental frequency of the IFFM signal to male standards (127Hz) to encompass extra information within the lower frequency range of aidable residual hearing in bimodal users. Both noises were checked on spectral comparability and scaled to the same root-mean-square (RMS) level.

### Set-up

**Fig 3:**
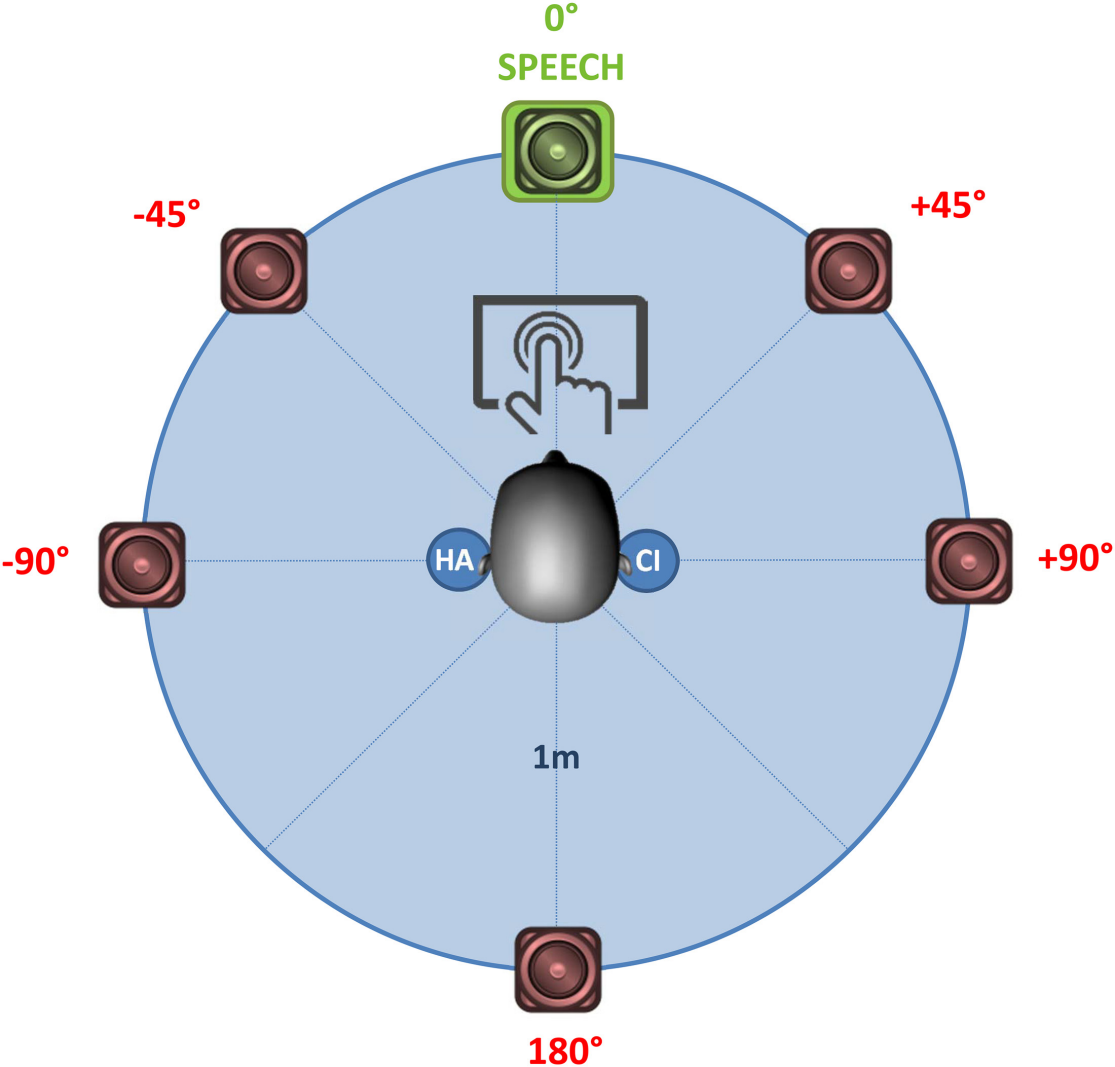
Test set-up. Six loudspeakers were positioned in a circle of 1m around the participant, who was fitted with a cochlear implant (CI) and hearing aid (HA) in opposite ears. Speech was always presented in front (0°) while noise was simultaneously presented from the other five speaker locations. Participants used a touchscreen in front to self-administer all tests.

Testing was performed in a sound-attenuated booth using a desktop computer and the Oldenburg measurement applications (OMA) software package (HörTech gGmbH, Oldenburg, Germany). Participants used a touchscreen to self-administer all tests. Sounds were directed via analog lines of two externally connected Multiface II^™^ soundcards (Hammerfall DSP System, RME, Audio AG, Haimhausen, Germany). The participant was seated amid an array of 6301B3X loudspeakers (Fostex, Tokyo, Japan) with a radius of one meter (Fig 3). Speech was presented in front (0°) while noise was continuously played from five surrounding speakers (+-45°, +-90°, 180°). By applying a fixed randomly generated phase delay to the same basic signal in each of the five masker channels, the surrounding noise was emitted in an uncorrelated manner. This resulted in a diffuse interference field, representative of a challenging situation like a restaurant dinner. Each loudspeaker was first calibrated individually for the same presentation level. Afterwards an overall adjustment was applied to the five masker channels to reach the desired calibration level for the total noise field.

### Sample size calculation

Sample size estimation for the primary outcome of speech intelligibility was based on data available from literature. The monaural beamformer under investigation has been observed to provide an improvement of 5.2 up to 5.6 dB SNR with a standard deviation of 0.7 up to 1.7 dB SNR [28,29] compared to an omnidirectional microphone (in or behind the pinna) in unilateral CI users. While the effect of asymmetry has not yet been investigated for beamforming in CI recipients, there are some studies in hearing aid users which have demonstrated a significant benefit of a symmetric over an asymmetric setting with an improvement of 1.9 up to 2.4 dB SNR [30,59,60]. To be able to detect the smallest primary effect, the estimated effect size within this study was set at 1.1 (i.e. 1.9 dB/1.7 dB). The required sample size was calculated for a paired samples statistical test using the statistical software G*Power 3.1.9 [61]. The power analysis indicated that a sample of 11 (parametric) up to 12 (non-parametric) subjects was required to attain a power of 80%. Based on these prospective calculations sample size was set at 12.

### Statistical analysis

The outcome data were inspected for missing values. One-way random intraclass correlation coefficients (ICC) [62] were obtained to evaluate the reliability of the outcome measures. Normality was checked by the Shapiro-Wilk test and visual inspection of the outcome distributions using histograms and Q-Q plots.

A two-way repeated measures analysis of variance (ANOVA) was conducted to investigate the influence of two factors, namely directionality and noise type, on speech intelligibility. The factor directionality included three levels (omni, asymmetric, symmetric) and the factor noise type consisted of two levels (stationary, fluctuating). Listening effort ratings were compared across three factors by a three-way repeated measures ANOVA: the factors directionality and noise type were the same as for speech intelligibility outcomes while the third factor refers to three tested levels (SRT, SRT+5 or SRT+10). To correct against sphericity violations a Greenhouse-Geisser adjustment was applied. Where statistically significant effects were identified, post-hoc comparisons were performed with two-tailed paired samples *t*-tests. Mean pairwise differences are presented accompanied by the standard error (SE). An alpha value of 0.05 was considered with a Bonferroni adjustment for multiple comparisons.

## Results

### Speech intelligibility

Individual SRT outcomes for all bimodal participants are presented (S1 Table). No missing data were ascertained. Only one valid outcome could be obtained for one of the six tested conditions for six of the participants. For all the other test conditions and for all the other participants the mean of the two outcomes was taken. When both outcomes for test and retest were available, the ICC for the average outcome as well as for a single measure was found to lie between 0.72 and 0.95, indicating a substantial to almost perfect reliability [63] of the outcome measure in all conditions.

**Fig 4:**
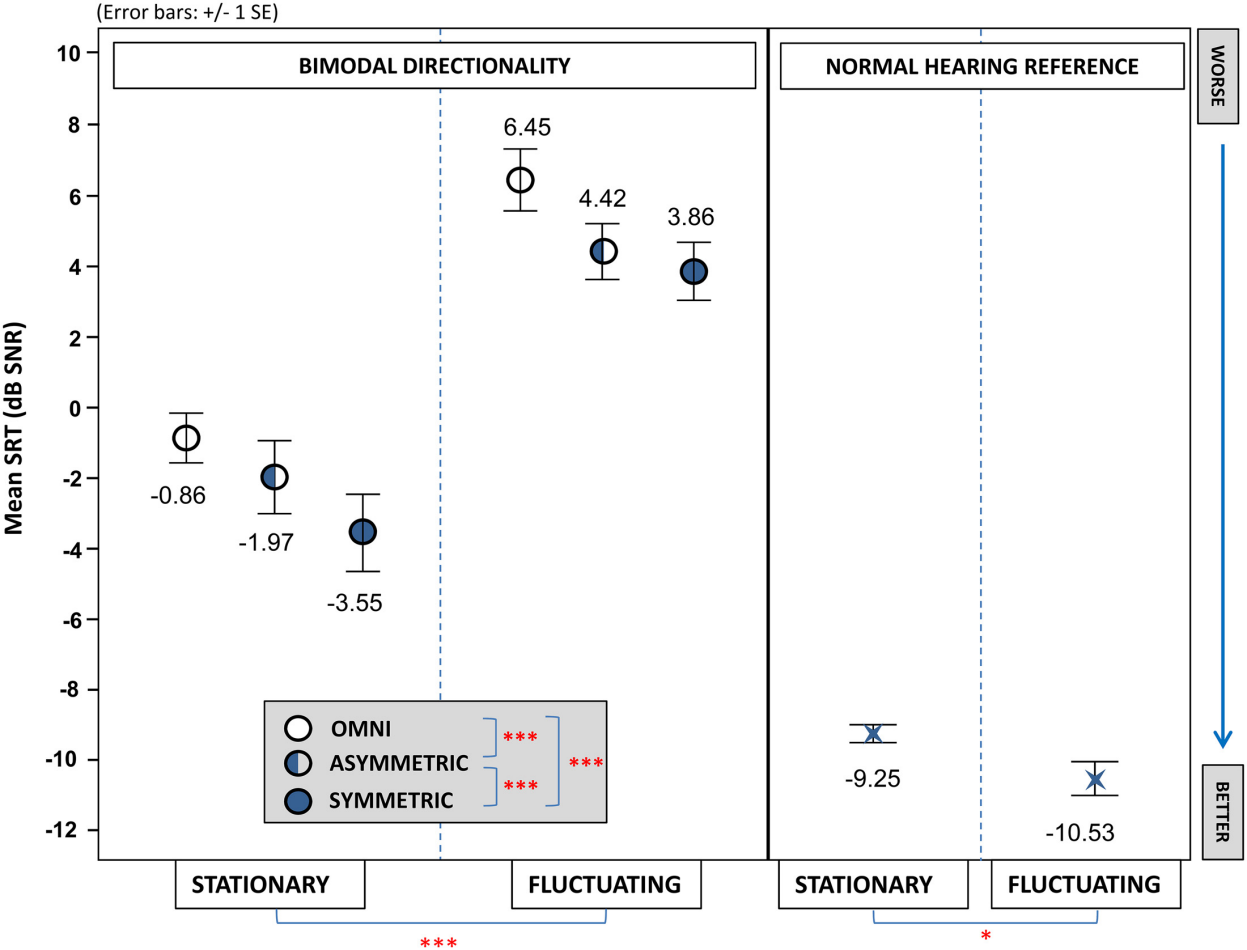
Speech intelligibility outcomes. Mean speech reception thresholds (SRT) in noise of the bimodal study group are presented for the six tested conditions. Each condition consisted of a directionality configuration (omnidirectional, asymmetric, symmetric) assessed within two different types of noise (stationary, fluctuating). For comparison, mean speech intelligibility scores of a normal hearing reference group tested in the same set-up are shown. A lower SRT-value represents a better outcome. Significant differences between test conditions are flagged (*p<0.05, **p<0.01, ***p<0.001).

A graphic presentation of the average bimodal SRT outcome across all test conditions is shown alongside the average results of a normal hearing reference group in both noise types (Fig 4). Data was normally distributed. In the bimodal study group, the two-way ANOVA indicated that both the main effects of directionality [F(1.40,15.38) = 50.30, p<0.001, ɳ _p_^2^ = 0.82] and noise type [F(1.00,11.00) = 237.13, p<0.001, ɳ _p_^2^ = 0.96] were highly significant. There appeared to be no interaction effect between directionality and noise type. Post-hoc comparisons demonstrated speech intelligibility to be significantly better for the asymmetric setting compared to the omnidirectional reference with a difference of 1.57±0.20 dB SNR (p<0.001). Symmetric directionality provided an additional significant improvement of 1.07±0.23 dB SNR (p<0.001) compared to the asymmetric configuration. Overall this resulted in a significant difference of 2.64±0.34 dB SNR (p<0.001) between symmetric directionality and the omnidirectional reference.

The post-hoc comparison between noise types showed that SRT outcomes were significantly higher in fluctuating noise compared to stationary noise (7.04±0.46 dB SNR (p<0.001)). In the normal hearing reference group, however, speech intelligibility proved to be slightly better in fluctuating compared to stationary noise with a significant difference of 1.28±0.46 dB SNR (p = 0.03).

### Listening effort

Individual listening effort ratings for all bimodal participants are listed in S2 Table. No missing data occurred. The ICC for the average across five ratings was found to lie between 0.64 and 0.95, indicating a substantial to almost perfect reliability [63] of the listening effort measurement in all conditions.

**Fig 5:**
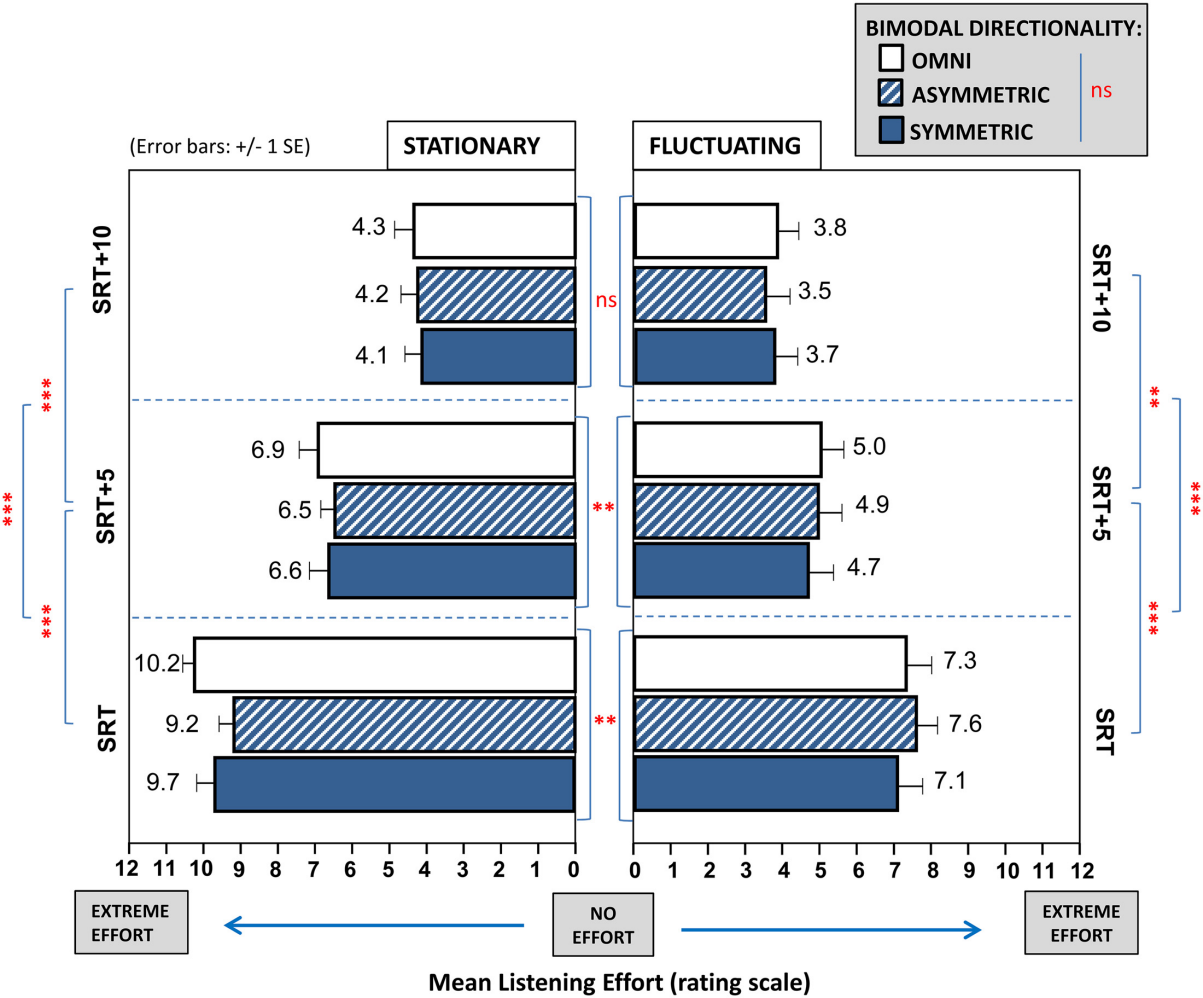
Listening effort outcomes. Mean listening effort ratings on a scale of 0 (‘no effort ‘) to 12 (‘extreme effort’) for the bimodal study group are presented for six test conditions at three levels. Test conditions consisted of a directionality configuration (omnidirectional, asymmetric beamforming, symmetric beamforming) assessed for two different noise types (stationary, fluctuating). Tested levels of SRT, SRT+5 and SRT+10 are expressed as levels relative to the participant’s individual speech-reception threshold (SRT) on the speech intelligibility task in the corresponding condition. Significant differences between test conditions are flagged (*p<0.05, **p<0.01, ***p<0.001). Ns = not significant.

Mean ratings of bimodal listening effort across all test conditions are presented (Fig 5). Data was normally distributed. The three-way ANOVA showed no significant main effects. There was also no interaction between directionality and noise type and level; directionality and noise type; and directionality and level. There was an interaction between noise type and level [F(1.53,16.88) = 10.42, p = 0.002, ɳ _p_^2^ = 0.49]. Therefore the simple effects of those two factors were further investigated. The results showed that listening effort was rated significantly higher at the participant’s SRT (9.68±0.34 points) when compared to SRT+5 (6.65±0.38 point) and SRT+10 (4.22±0.39 points) in stationary noise (all p<0.001). The same order of level effect was observed in fluctuating noise comparing the rated effort at SRT (7.31±0.57 points), SRT+5 (4.87±0.57 points) and SRT+10 (3.69±0.55 points) (all p<0.002). Listening effort was assigned a significantly lower rate in fluctuating when compared to stationary noise at SRT (difference 2.38±0.55 points, p = 0.001) and SRT+5 (difference 1.78±0.56 points, p = 0.008) but not at SRT+10 (difference 0.53±0.31 points, p = 0.11).

## Discussion

### Summary of findings

Application of a monaural beamformer in bimodal CI-recipients improved speech intelligibility in stationary as well as fluctuating noise. Asymmetric directivity provided an average benefit of 1.6 dB compared to the omnidirectional standard. Symmetric directivity revealed an additional benefit of 1.1 dB, leading to an overall improvement of 2.6 dB. Listening effort decreased with increasing SNR but did not show an effect of directivity on top of speech intelligibility. Overall, bimodal users performed about 7 dB SNR worse in fluctuating as compared to stationary noise, while more listening ease was reported.

### Speech intelligibility

#### Degree of directional benefit

The effect of the investigated monaural beamformer has been previously reported to be 3.7 up to 5.6 dB in unilateral CI-recipients [28,29]. Compared to those findings, the unilateral degree of benefit in this study is rather small (1.6 dB) although significant. It is known that the benefit of directional systems is dependent on the used listening test set-up [17]. The current test set-up included speakers up to ±45 degrees, which still fell within the frontal beam of this monaural beamformer according to its polar plot [29]. The demanding spatial setting therefore is thought to be the primary reason for the found degree of directional benefit. To a smaller extent also the used reference condition could have played a role. Directivity in the CI-ear namely was compared to the reference of an in-the-concha microphone, already giving a first degree of natural directionality [49] and leaving less room for improvement through beamforming [25,28]. Withal, compared to results of unilateral CI-studies [28,29], it should also be emphasized that this study evaluated bimodal listeners. Although the benefit of bimodal hearing was not separately assessed, it is known that bimodal hearing can provide significant bilateral and binaural benefits [4–6,10–12]. There is thus less room for improvement by beamforming algorithms when listening binaurally [64], probably explaining the smaller degree of directional benefit found in this study.

#### Effect of (a)symmetric directivity

To date asymmetric directivity has only been suggested in bilateral HA-users as a permanent setting instead of manually switching between directional modes [41,65]. According to the “better SNR ear” principle, the effective SNR should be at least as good as the better of the two ears [66]. But the reported difference between asymmetric and symmetric directivity varies across HA-studies, ranging from no difference [41,65,67,68] to a significant benefit in favor of the symmetric configuration [30,59,60]. However, CI-recipients are a different population, especially in the event of bimodal fitting. In bimodal users an asymmetric situation is often predetermined, having a CI in one ear and a HA in the other. The current study is the first to assess (a)symmetric directivity within a group of bimodal listeners. As demonstrated by the CNC scores in quiet (Table 1), the CI-ear was the primary speech input for most participants. Therefore only the CI was put in directional mode in the asymmetric setting. Results revealed a substantial improvement of the symmetric relative to the asymmetric configuration. Symmetric directivity (2.6 dB) almost doubled the advantage of asymmetric directivity (1.6 dB). Although no studies are known to evaluate the effect of (a)symmetric directivity within bimodal or bilateral CI-recipients, a recent study [64] did point towards the benefit of symmetric directivity when comparing groups. They tested both bimodal and bilateral CI-users on speech intelligibility in noise with and without a monaural adaptive beamformer activated in their CI(s). The SRT improvement was found to be twice as high in the bilateral group as in the bimodal group, demonstrating the benefit of a bilateral combination of beamforming. A set-up with a moving noise source in the back hemisphere and the bilateral directivity benefit was linked to the effect of head-shadow. However, the current study used a fixed set-up. It may therefore be that the effects of summation (combining two comparable inputs) and complementarity (combining two inputs with access to supplemental information [69]) played a major role in the demonstrated symmetric benefit in this bimodal population.

#### Effect of noise type

In contrast to stationary noise, the fluctuating modified IFFM noise contained temporal gaps, and although non-intelligible, resembled a single speaker possibly inducing informational masking. A normal hearing reference group scored about 1.3 dB better in fluctuating when compared to stationary noise. By listening “in the noise gaps”, normal hearing listeners are known to benefit from a masking release up to 7 dB [31]. The degree of normal hearing masking release in this realistic set-up was smaller, probably because temporal fluctuations were reduced by presenting multiple uncorrelated spatially separated sources simultaneously. The average broad band modulation depth of the fluctuating masker (calculated according to IEC 60118–15) was 7 dB for the five-talker signal compared to 17 dB for the single talker signal. For reference, the modulation depth of the stationary noise was 2 dB albeit single or multi sourced.

In contrast to the normal hearing reference, bimodal listeners were not only unable to benefit from available gaps, they experienced a detrimental effect (7 dB) from the fluctuating masker when compared to stationary noise. This adverse effect, which has been previously reported for CI processing, is mainly caused by technical and physiological properties of CI stimulation [70] resulting in a limited frequency and temporal resolution [71–73]. Furthermore IFFM-like signals are known to have a distracting effect [31] which could result in a harder segregation task and thus demanding more attentional load [74].

The noise reduction strategy available in the investigated CI processor could also have had an impact. This algorithm is known to improve speech intelligibility in stationary noise [46,47] but has a smaller benefit in fluctuating noise [75]. This could have magnified the difference in SRT outcomes between the two noise types. The participants’ daily setting of this algorithm was adopted in the test processor, resulting in different settings across participants. This between-subject factor was not included in the analysis due to the small sample size.

A study that compared a fixed and an adaptive directional microphone in CI-patients reported on average more directional benefit in fluctuating multi-talker babble noise as compared to a steady-state speech-weighted noise [24]. Concerning the benefit of directional microphones there might be an interaction with the masker type used to test the difference in speech recognition performance. The current study however cannot support this earlier finding, since no interaction between directionality and noise type was detected.

### Listening effort

#### Dimension on top of speech intelligibility

Literature shows that listening effort is an additional dimension next to speech intelligibility and should be included when evaluating in noisy listening conditions [35,36]. In the current study a quick and clinically applicable subjective rating task was included to assess listening effort independent of individual speech intelligibility [76]. Tested levels were therefore defined relative to the participant’s SRT instead of conventionally testing at fixed SNRs. Given the slope of the optimized Matrix speech material in stationary noise for normal hearing subjects (13.7%/dB) [52], tested SRT levels are assumed to correspond to 50% intelligibility (SRT) and go up to around 100% intelligibility for SRT+5 and SRT+10. It is known that the ease of listening increases with increasing SNR levels [76,77]. Results did support this since effort was rated significantly lower at SRT+10 compared to SRT+5 and SRT for both noise types. But even at a level of SRT+10, the effort rated by bimodal participants did not drop to zero. This finding suggests that severe hearing impaired listeners may never report listening to be implicitly easy, supporting hypotheses linking hearing difficulties to an increased cognitive processing load [74].

#### Effect of (a)symmetric directivity

A reduction in effort through the application of directional fitting in HA-users has previously been reported in literature [41,42]. However by testing at fixed SNRs, earlier studies did not correct for speech intelligibility performance [42]. A recent study in older HA listeners did report a reduction in effort in a dual task paradigm when using directional microphones at a fixed intelligibility level, even though no significant difference between objective and self-reported ratings of listening effort was found [78].

In the current bimodal study, levels relative to the participant’s SRT were tested and no effect of directionality on the degree of listening effort was found. The observed directional benefit for speech intelligibility without additional effect on rated effort supports the statement that microphone directionality created an unadulterated SNR improvement. This result also demonstrated that the independent across ear operation of a monaural beamformer applied bilaterally did not seem to cause confounding cues.

#### Effect of noise type

Subjective effort rating has previously proven to be sensitive to differences in noise type [79]. Results show that effort was rated lower in fluctuating noise than in stationary noise at SRT and SRT+5, but not at SRT+10. At SRT+10, it could be expected that speech surpasses the noise and thus the basic effort of speech intelligibility is measured rather than noise influence. The effect of noise type on lower SNRs seems somewhat contra-intuitive since performance in fluctuating noise was significantly worse while effort was rated to be easier. Both speech intelligibility and listening effort are correlated with SNR, but they are known to be two different factors related in a non-linear manner [36]. The current results suggest that this relationship between intelligibility and listening effort is quite different for stationary and fluctuating noise. It seems that listening effort is closely related to the physically presented SNRs. Since levels were presented relative to the participant’s SRT, SNR levels for fluctuating noise were up to 7 dB higher when compared to stationary noise, which was reflected in lower effort ratings. Furthermore also other unknown factors may have influenced this outcome. It has for example been suggested that the individual differences in working memory capacity may influence the relative perceived effort in different types of noise [77]. A study in young normal hearing adults showed that cognitive spare capacity performance was disrupted more in steady-state than in speech-like noise, possibly because selective attention could be used to ignore the speech-like background [80]. In general the found difference of noise type is supported by an earlier study in normal hearing, mild and moderately hearing impaired listeners that reported listening effort to be less in cafeteria noise compared to stationary noise, while intelligibility was greater in the latter [76,81].

### Challenges and future outlook

Hearing aid manufacturers have been providing their devices with directional microphone systems for years. Likewise all of the major CI manufacturers implement a beamforming solution in their current speech processors. Therefore most bimodal recipients nowadays have access to a directional microphone system in both devices and thus comparable benefits as the ones observed in the current study are to be expected. However the long-term use of bimodal directivity in a variety of daily situations has not yet been addressed. Also the impact of the fact that most bimodal listeners make use of hearing systems giving access to different directional systems in both ears is still unknown. Even if the same beamforming system is available in both CI and HA, automatic program selection, which nowadays still operates independently per device, could cause the devices to select different settings at different scenes instead of a symmetric operation. The real-time and real-world application should therefore be the topic of further investigation and efforts should be made to enhance inter-device communication between CI and HA-systems. If such a bimodal communication system becomes available, also a binaural beamformer could be applied to even further improve directionality for bimodal listeners as has been proven for bilateral HA [82] and bilateral CI-recipients [29].

## Supporting Information

S1 Table Individual speech reception thresholds in noise (dB SNR). Mean of two outcomes except ^1^ based on single outcome when other outcome ^a^ outside range of presented SNR's or ^b^ larger than 15dB SNR.
(XLSX)

S2 Table Individual listening effort ratings (scale 0–12). Mean of five ratings on scale 0 (no effort) to 12 (extreme effort).
(XLSX)
